# Nonreciprocal broken ray transforms with applications to fluorescence imaging

**DOI:** 10.1088/1361-6420/aacec7

**Published:** 2018-07-11

**Authors:** Lucia Florescu, Vadim A Markel, John C Schotland

**Affiliations:** 1 Centre for Vision, Speech and Signal Processing, University of Surrey, GU2 7XH, United Kingdom; 2 Department of Radiology, University of Pennsylvania, Philadelphia, PA 19104, United States of America; 3 Department of Mathematics and Department of Physics, University of Michigan, Ann Arbor, MI 48109, United States of America; l.m.florescu@surrey.ac.uk; vmarkel@pennmedicine.upenn.edu; schotland@umich.edu

**Keywords:** Broken ray transform, star transform, fluorescence imaging, Compton scattering

## Abstract

Broken ray transforms (BRTs) are typically considered to be reciprocal, meaning that the transform is independent of the direction in which a photon travels along a given broken ray. However, if the photon can change its energy (or be absorbed and re-radiated at a different frequency) at the vertex of the ray, then reciprocity is lost. In optics, non-reciprocal BRTs are applicable to imaging problems with fluorescent contrast agents. In the case of x-ray imaging, problems with single Compton scattering also give rise to non-reciprocal BRTs. In this paper, we focus on tomographic optical fluorescence imaging and show that, by reversing the path of a photon and using the non-reciprocity of the data function, we can reconstruct simultaneously and independently all optical properties of the medium (the intrinsic attenuation coefficients at the excitation and the fluorescence frequency and the concentration of the contrast agent). Our results are also applicable to inverting BRTs that arise due to single Compton scattering.

## 1. Introduction

The broken-ray transform (BRT), also referred to as the V-line transform, has attracted significant recent attention [1–9]. This transform is a generalization of the classical Radon transform to the physical setting in which a photon can change its propagation direction due to scattering. A related recent development is inversion of the conical Radon transform, which arises in tomographic applications of the Compton camera [10–18], although in the latter case the vertex of the V-line is located at the detection surface rather than within the medium.

The BRT is defined in terms of integrals of a function (the attenuation coefficient) along two rays with a common vertex. The ray directions are fixed and determined by the directions of the source and detector. However, the position of the vertex can be scanned over the plane that contains both rays. The corresponding data provides sufficient information to reconstruct the attenuation coefficient in the plane thus defined, assuming that the scattering coefficient is spatially homogeneous. An interesting feature of the BRT is that its inversion does not require data from multiple projections; a single scan, analogous to one plane-parallel projection of traditional x-ray tomography, suffices for reconstruction [19]. In the single-projection geometry, inversion of the BRT is a mildly ill-posed problem [20].

If the scattering coefficient of the medium is not spatially homogeneous, more elaborate methods can be employed to reconstruct the scattering and the attenuation coefficients separately. This is possible in a measurement geometry with several (more than two) rays having a common vertex and lying in the same plane [21]. A direct and stable inversion formula for a transform involving four such rays has been derived in [22]. The formula is local, that is, it involves only first-order derivatives with respect to the vertex position, and therefore it does not require a ‘complete’ data set. This property is very useful and, in principle, is unattainable in transforms involving only integrals along lines. A generalization of the local inversion formula to the case of an arbitrary number of rays, which defines the star transform, was derived in [23]. In particular, it was shown that a local formula can be obtained with only three rays. This imaging geometry is considered below. We note that a single ‘star’ consists of several distinct broken rays, some of which can share one edge. In a given measurement (utilizing a fixed source-detector pair), a photon always travels along one of the broken rays. We can utilize several detector arrays to perform measurements corresponding to different broken rays belonging to the same ‘star’ in one scan.

In previously considered applications, the BRT is reciprocal, which means that the transform is independent of the direction in which the photon travels along a given broken ray. Consequently, the associated measurements are independent of the interchange of the source and detector. However, if the photon is scattered or absorbed and re-emitted at a different frequency, the reciprocity of the BRT is lost. In this paper, we consider a non-reciprocal BRT applicable to the problem of fluorescence imaging in a weakly-scattering or non-scattering medium. In this case, the photon travels along a line until it is absorbed by a fluorophore molecule and then is re-emitted in a different direction and at a different (generally lower) frequency. We show that the non-reciprocity of the BRT allows us to access additional information about the medium by interchanging the source and detector in each pair. In particular, this approach allows us to reconstruct the attenuation coefficient of the medium at both the excitation and the fluorescence frequencies independently, as well as the concentration of the fluorophore. The method described in this paper requires doubling the number of physical measurements, but it does not involve any assumptions about the spectral dependence of the attenuation coefficient, which can generally differ at different points in the medium.

The proposed method is also applicable to x-ray imaging where the photon can change its direction due to Compton scattering. As is well known, the latter is accompanied by a reduction of the photon energy. Since the attenuation coefficient can depend on energy, this poses an additional challenge. This problem was solved in [24] under the assumption that the spectral dependence of the attenuation coefficient is linear. Here we show that accounting for the non-reciprocity of the measurements allows one to avoid making assumptions about the spectral dependence of the attenuation. However, in this case, we are restricted to using only imaging geometries in which the scattering angles (and hence the Compton energy shifts) are the same. The three-ray star geometry considered below satisfies this requirement.

The remainder of this paper is organized as follows. In section 2, we consider the coupled transport equations and show that, in a non-scattering or a weakly-scattering medium, the specific intensity of light at the fluorescence frequency due to a collimated incident beam at the excitation frequency is mathematically related to a BRT of the medium. In section 3, we define the data function to be used in the reconstruction of the functions of interest. In section 4, we consider a more general arrangement of sources and detectors in which several broken rays with a common vertex form a ‘star’. In this section we show how the BRT non-reciprocity can be used to formulate the inverse problem. In section 5, we show how all three functions of interest (the background attenuation coefficients at the excitation and fluorescence frequencies and the concentration of fluorophore) can be reconstructed stably and separately by accounting for the BRT non-reciprocity. Explicit image reconstruction formulas are derived. In this section, we assume that the ‘complete data’ are available. In section 6, we consider physical situations in which the complete data are not available and sketch some approaches to performing partial or complete reconstructions in this less favorable case. Finally, section 7 contains a summary of the obtained results.

## 2. Coupled transport equations

We begin by considering the physical problem of fluorescence imaging in a weakly-scattering or non-scattering medium, taken to be a three-dimensional bounded domain Ω. In the absence of scattering, the transport equations describing the propagation of light at the excitation and fluorescent frequencies (distinguished by the subscripts *e* and *f*, respectively) are of the form

1*a* \begin{align*} \newcommand{\e}{{\rm e}} \displaystyle \label{RTE_e} \left[ \hat{\bf s} \cdot \nabla + \mu_e({\bf r}) \right] I_e({\bf r}, \hat{\bf s}) = 0 , \nonumber \end{align*}

1*b* \begin{align*} \newcommand{\e}{{\rm e}} \displaystyle \label{RTE_f} \left[ \hat{\bf s} \cdot \nabla + \mu_f({\bf r}) \right] I_f({\bf r}, \hat{\bf s}) = S({\bf r}, \hat{\bf s}) \ . \nonumber \end{align*}

Here $I_e({\bf r}, \hat{\bf s})$, $I_f({\bf r}, \hat{\bf s})$ are the specific intensities of light at the position ${\bf r}$ in the direction $\hat{\bf s}$, and $\mu_e$, $\mu_f$ are the attenuation coefficients of the medium at the frequencies indicated by the subscripts, and *S* is the fluorescent source. Equations (1*a*) and (1*b*) are also supplemented by the half-range boundary conditions

2 \begin{align*} \newcommand{\e}{{\rm e}} \displaystyle \label{BC} I_e({\bf r},\hat{\bf s}) = I_{\rm inc}({\bf r}, \hat{\bf s}) \ \ {\rm and} \ \ I_f({\bf r},\hat{\bf s}) = 0 \ \ \ {\rm for} \ \ \ \hat{\bf s}\cdot\hat{\bf n}({\bf r}) &lt; 0 , \quad {\bf r} \in \partial\Omega, \nonumber \end{align*}

where $\hat{\bf n}$ is the outward unit normal to $\partial\Omega$ and *I*_inc_ is the incident specific intensity at the excitation frequency. Note that no light at the fluorescent frequency enters the medium except due to the source *S*, which is defined below. For a medium containing fluorescent molecules of the number density $n({\bf r})$, we can write

3*a* \begin{align*} \newcommand{\e}{{\rm e}} \displaystyle \mu_e({\bf r}) = \mu^{(0)}_e({\bf r}) + \sigma_e n({\bf r}) , \nonumber \end{align*}

3*b* \begin{align*} \newcommand{\e}{{\rm e}} \displaystyle \mu_f({\bf r}) = \mu^{(0)}_f({\bf r}) + \sigma_f n({\bf r}) , \nonumber \end{align*}

where $\mu^{(0)}_e({\bf r})$ and $\mu^{(0)}_f({\bf r})$ are the intrinsic (background) attenuation coefficients of the medium and $\sigma_e$, $\sigma_f$ are the absorption cross sections of the fluorescent molecules at the frequencies indicated by the subscripts. The cross sections are assumed to be known from spectroscopic measurements and it is expected that $\sigma_f &lt; \sigma_e$. The source function at the fluorescence frequency is given by

4 \begin{align*} \newcommand{\e}{{\rm e}} \displaystyle S({\bf r}, \hat{\bf s}) = \frac{\eta \sigma_e}{4\pi} n({\bf r}) u_e({\bf r}) , \nonumber \end{align*}

where *η* is the quantum efficiency of the fluorophores and the energy density $u_e({\bf r}) = \int I_e({\bf r}, \hat{\bf s}) {\rm d}^2s$. If the incident light is injected into the medium as a narrow collimated beam, then it can be seen that [19]

5 \begin{align*} \newcommand{\e}{{\rm e}} \displaystyle \label{ue} u_e({\bf r}) = \frac{W}{\vert {\bf r} - {\bf r}_1 \vert^2} \exp\left( -\int_{{\bf r}_1}^{\bf r} \mu_e {\rm d}\ell \right) \delta\left( \hat{\bf s}_1 - \frac{{\bf r} - {\bf r}_1}{|{\bf r} - {\bf r}_1|}\right) \ . \nonumber \end{align*}

Here ${\bf r}_1$ and ${\bf s}_1$ are the location and direction of the source, *W* is the source power, and $ \newcommand{\e}{{\rm e}} \int_{\bf a}^{\bf b} f {\rm d}\ell$ is the integral of *f* along the line connecting the two points ${\bf a}$ and ${\bf b}$.

Let the specific intensity at the fluorescence frequency be measured at the point ${\bf r}_2 \in \partial\Omega$ and in the direction $\hat{\bf s}_2$. Then it follows from (1*b*) that

6 \begin{align*} \newcommand{\e}{{\rm e}} \displaystyle \label{If_1} I_f({\bf r}_2, \hat{\bf s}_2) = \frac{\eta \sigma_e W}{4\pi} \int {\rm d}^3r \frac{n({\bf r}) u_e({\bf r})}{\vert {\bf r}_2 - {\bf r} \vert^2} \exp\left( -\int_{\bf r}^{{\bf r}_2} \mu_f {\rm d}\ell \right) \delta\left( \hat{\bf s}_2 - \frac{{\bf r} - {\bf r}_2}{|{\bf r} - {\bf r}_2|}\right) \nonumber \end{align*}

where we have accounted for the isotropy of the source function *S*. An integral similar to (6) has been computed in the appendix of [19]. Here we adduce the final result,

7 \begin{align*} \newcommand{\e}{{\rm e}} \displaystyle \label{If_4} I_f({\bf r}_2, \hat{\bf s}_2) =&amp;\, \frac{\eta \sigma_e W}{4\pi r_{21} \sin\theta_1 \sin\theta_2} \exp\left( -\int_{{\bf r}_1}^{\bf R} \mu_e {\rm d}\ell - \int_{\bf R}^{{\bf r}_2} \mu_f {\rm d}\ell \right)\nonumber \\ &amp; \times n({\bf R}) \Theta(\pi - (\theta_1 + \theta_2)) \delta(\varphi_2 - \pi) \ . \nonumber \end{align*}

**Figure 1.**
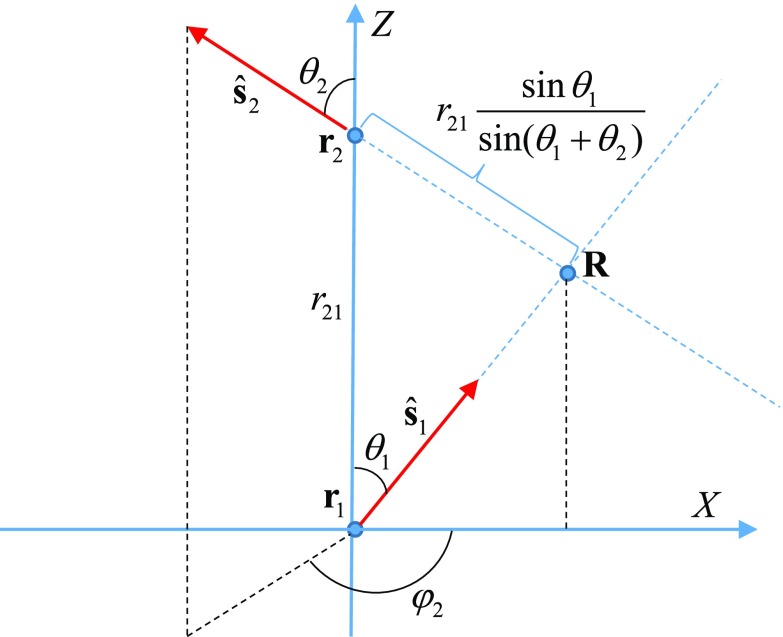
Illustrating the geometry relevant to equation (8).

Various geometrical quantities and objects appearing in (7) are illustrated in figure 1. In particular, ${\bf r}_{21} = {\bf r}_2 - {\bf r}_1$, ${\bf R} = {\bf r}_2 - r_{21} \frac{\sin\theta_1}{\sin(\theta_1 + \theta_2)} \hat{\bf s}_2$ is the position of the vertex, that is, the point where the two rays shown in figure 1 by the dashed blue lines intersect, $\Theta(\cdot)$ is the unit step function, and we have assumed that the vectors ${\bf r}_{21}$ and $\hat{\bf s}_1$ lie in the *XZ* plane of the laboratory frame. The delta-function $\delta(\varphi_2 - \pi)$ ensures that $\hat{\bf s}_2$ lies in the same plane. Therefore, equation (7) yields a nonzero result only if the three vectors ${\bf r}_{21}$, $\hat{\bf s}_1$ and $\hat{\bf s}_2$ lie in the same plane, which defines the slice of the medium in which the two-dimensional reconstruction of $\mu^{(0)}_e({\bf r})$, $\mu^{(0)}_f({\bf r})$ and $n({\bf r})$ is performed. Finally, the function $\Theta(\pi - \theta_1 + \theta_2)$ is equal to unity if the vertex exists and to zero otherwise. We note that, when experimental data are used, accounting for the geometrical factor $1/r_{21} \sin\theta_1\sin\theta_2$ in (7) is important for correct formulation of the inverse problem.

## 3. Data function

Our goal is to reconstruct the three functions $\mu^{(0)}_e({\bf r})$, $\mu^{(0)}_f({\bf r})$ and $n({\bf r})$ separately from collimated boundary measurements of the fluorescence intensity. In an experiment, the latter can be registered with the use of a spectral filter that excludes all radiation at the excitation frequency. To proceed, it is convenient to introduce the dimensionless concentration of fluorophores according to $ \newcommand{\e}{{\rm e}} \tilde{n}({\bf r}) = \eta \sigma_e^{3/2} n({\bf r})$. We note that $\tilde{n} \sim 1$ corresponds to a very high concentration and, in practice, we expect that $\tilde{n}\ll 1$ will hold. Let us assume that the condition $\theta_1+\theta_2&lt;\pi$ holds so that the vertex exists. We then have

8 \begin{align*} \newcommand{\e}{{\rm e}} \displaystyle \label{If_5} \frac{\sqrt{\sigma_e}r_{21}}{W} I_f({\bf r}_2, \hat{\bf s}_2) = \frac{\tilde{n}({\bf R})}{4\pi \sin\theta_1 \sin\theta_2}\exp\left( -\int_{{\bf r}_1}^{\bf R} \mu_e {\rm d}\ell - \int_{\bf R}^{{\bf r}_2} \mu_f {\rm d}\ell \right) \delta(\varphi_2 - \pi) \ . \nonumber \end{align*}

Both sides of this equation are dimensionless. However, the expression is still singular and not amenable to direct interpretation as a measurable signal. To alleviate this problem, we note that the fluorescence intensity at the point of observation ${\bf r}_2$ depends on the spherical coordinates of the unit vector $\hat{\bf s}_2$, $\theta_2$ and $\phi_2$. The dependence on $\theta_2$ is slow ($1/\sin\theta_2$) while the dependence on $\varphi_2$ is fast and expressed mathematically by the delta-function $\delta(\varphi_2 - \pi)$. On the other hand, all physical detectors measure the specific intensity in some finite solid angle. We can assume therefore that the measured quantity is $\int_{\pi-\delta}^{\pi+\delta} I_f {\rm d}\varphi_2$, where *δ* is a small angle.

We can now define the data function $\phi_{12}({\bf R})$ as follows:

9 \begin{align*} \newcommand{\e}{{\rm e}} \displaystyle \label{phi_def} \phi_{12}({\bf R}) = -\log \left[4\pi \sin\theta_1 \sin\theta_2 \frac{\sqrt{\sigma_e}r_{21}}{W} \int_{\pi-\delta}^{\pi+\delta}I_f({\bf r}_2, \hat{\bf s}_2) {\rm d}\varphi_2 \right] \ . \nonumber \end{align*}

Let $\hat{\bf s}_1$ and $\hat{\bf s}_2$ be fixed and such that the vertex exists. Then there is a one-to-one correspondence between the position of the vertex ${\bf R}$ and the pair of variables $({\bf r}_1$, ${\bf r}_2$). In what follows, we will view ${\bf R}$ as the independent variable. Applying the definition (9) to (8), we obtain the equation

10 \begin{align*} \newcommand{\e}{{\rm e}} \displaystyle \label{eq_12} I_{e,1}({\bf R}) + I_{f,2}({\bf R}) + \xi({\bf R}) = \phi_{12}({\bf R}) , \nonumber \end{align*}

where

11*a* \begin{align*} \newcommand{\e}{{\rm e}} \displaystyle \label{eta_def} \xi({\bf R}) = -\log[\tilde{n}({\bf R})] , \nonumber \end{align*}

11*b* \begin{align*} \newcommand{\e}{{\rm e}} \displaystyle I_{e,1}({\bf R}) = \int_0^\infty \mu_e({\bf R} - \hat{\bf s}_1 \ell) {\rm d}\ell , \nonumber \end{align*}

11*c* \begin{align*} \newcommand{\e}{{\rm e}} \displaystyle I_{f,2}({\bf R}) = \int_0^\infty \mu_f({\bf R} + \hat{\bf s}_2 \ell) {\rm d}\ell \ . \nonumber \end{align*}

Here we have assumed that $\mu_e({\bf r})$ and $\mu_f({\bf r})$ are supported in Ω so that the ray integrals can be extended to infinity.

Note that the data function can be measured only for such positions of the vertex ${\bf R}$ that $\tilde{n}({\bf R}) \neq 0$. If $\tilde{n}({\bf R}) = 0$, the measured fluorescence intensity is zero, at least, within the approximation used here, and the logarithm in (9) is undefined.

## 4. Inverse problem

**Figure 2.**
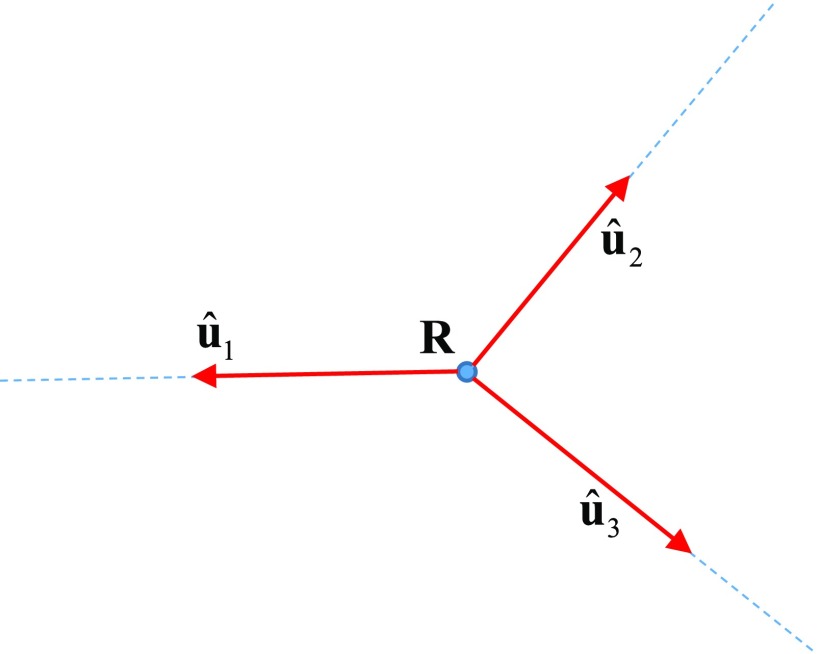
The imaging geometry for the special case of three rays.

Above, we have considered one broken ray defined by the locations of the source and detector (${\bf r}_1$ and ${\bf r}_2$) and the location of the vertex ${\bf r}$ (from now on, we denote the vertex position by the small letter ${\bf r})$. We now allow a more general arrangement of source-detector pairs, assuming that all broken rays corresponding to different source-detector pairs have the same vertex and that this vertex can be scanned with a reasonable spatial resolution over the sub-region of Ω in which $\tilde{n}({\bf r}) &gt; 0$. Some of the broken rays in a given ‘star’ can have a common edge. The imaging geometry is sketched in figure 2, where $\hat{\bf u}_k$ are unit vectors pointing from the vertex to a source or detector. We note that $\hat{\bf u}_k = \pm \hat{\bf s}_k$, where the plus sign must be chosen if the ray points towards a detector and the minus sign is chosen if the ray points towards a source.

The generalization of (10) to the above case is the *N*-ray star transform equation

12 \begin{align*} \newcommand{\e}{{\rm e}} \displaystyle \label{eq_kl} I_{e,k}({\bf r}) + I_{f,l}({\bf r}) + \xi({\bf r}) = \phi_{kl}({\bf r}) , \ \ \ k,l=1,\ldots , N , \ \ \ k\neq l \ . \nonumber \end{align*}

Here

13*a* \begin{align*} \newcommand{\e}{{\rm e}} \displaystyle I_{e,k}({\bf r}) = \int_0^\infty \mu_e({\bf r} + \hat{\bf u}_k \ell){\rm d}\ell , \nonumber \end{align*}

13*b* \begin{align*} \newcommand{\e}{{\rm e}} \displaystyle I_{f,k}({\bf r}) = \int_0^\infty \mu_f({\bf r} + \hat{\bf u}_k \ell){\rm d}\ell \ . \nonumber \end{align*}

Notably, the data function $\phi_{kl}({\bf r})$ is not symmetric with respect to the interchange of the indexes *k* and *l*. Physically, this means that the measurements do not obey reciprocity under the interchange of the source and the detector. The lack of reciprocity follows from the spectral dependence of the attenuation, that is, from $\mu_e({\bf r}) \neq \mu_f({\bf r})$. This is different from the star transform that arises in single-scattering tomography [23]. In the latter case, the data function is symmetric. In the case considered here, the lack of symmetry of the data function allows one to derive a local reconstruction algorithm, similar to those described in [22, 23], which yields all three functions $\mu_e({\bf r})$, $\mu_f({\bf r})$ and $\tilde{n}({\bf r})$. Let us consider the symmetric and anti-symmetric linear combinations of the data,

14 \begin{align*} \newcommand{\e}{{\rm e}} \displaystyle \label{phi_plus_minus} \phi_{kl}^{(+)}({\bf r}) = \frac{1}{2}\left[\phi_{kl}({\bf r}) + \phi_{lk}({\bf r}) \right] , \ \ \phi_{kl}^{(-)}({\bf r}) = \phi_{kl}({\bf r}) - \phi_{lk}({\bf r}) \ . \nonumber \end{align*}

It should be kept in mind that measuring these linear combinations requires reversing the path of the photon, that is, physically interchanging the source and detector for each broken ray or, alternatively, performing an additional scan with interchanged source and detector arrays. From (12) and (14), we find the following set of linear equations:

15*a* \begin{align*} \newcommand{\e}{{\rm e}} \displaystyle \label{eq_kl_p} I_{k}^{(+)}({\bf r}) + I_{l}^{(+)}({\bf r}) + \xi({\bf r}) = \phi_{kl}^{(+)}({\bf r}) , \nonumber \end{align*}

15*b* \begin{align*} \newcommand{\e}{{\rm e}} \displaystyle \label{eq_kl_m} I_{k}^{(-)}({\bf r}) - I_{l}^{(-)}({\bf r}) = \phi_{kl}^{(-)}({\bf r}) , \nonumber \end{align*}

where

16*a* \begin{align*} \newcommand{\e}{{\rm e}} \displaystyle \label{star_p} I_{k}^{(+)}({\bf r}) = \int_0^\infty \bar{\mu}({\bf r} + \hat{\bf u}_k \ell) {\rm d}\ell , \nonumber \end{align*}

16*b* \begin{align*} \newcommand{\e}{{\rm e}} \displaystyle \label{star_m} I_{k}^{(-)}({\bf r}) = \int_0^\infty \Delta({\bf r} + \hat{\bf u}_k \ell) {\rm d}\ell , \nonumber \end{align*}

and

17*a* \begin{align*} \newcommand{\e}{{\rm e}} \displaystyle \bar{\mu}({\bf r}) = \frac{1}{2}\left[ \mu_e({\bf r}) + \mu_f({\bf r}) \right] , \nonumber \end{align*}

17*b* \begin{align*} \newcommand{\e}{{\rm e}} \displaystyle \Delta({\bf r}) = \mu_e({\bf r}) - \mu_f({\bf r}) \ . \nonumber \end{align*}

## 5. Inversion with complete data

As mentioned in section 3, the data functions $\phi_{kl}({\bf r})$ or $\phi_{kl}^{(\pm)}({\bf r})$ are defined only for positions of the vertex ${\bf r}$ such that $\tilde{n}({\bf r}) &gt; 0$. Otherwise, the data function can not be measured. In practice, the condition that the data is measurable is stronger and reads $\tilde{n}({\bf r}) \geqslant \tilde{n}_{\rm min}({\bf r}) &gt; 0$. Here $\tilde{n}_{\rm min}({\bf r})$ is experimentally determined.

**Figure 3.**
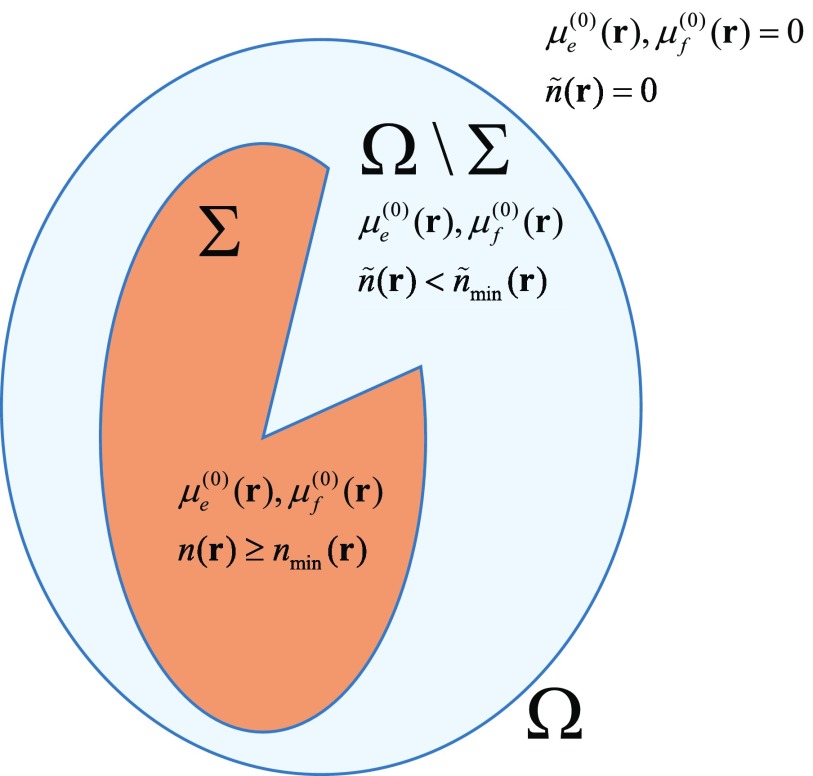
Illustrating the domains in which image reconstruction is performed.

The above considerations are illustrated in figure 3. Let $\Sigma \subset \Omega$ be the region in Ω in which the fluorescence signal can be measured. Σ can be defined with or without reference to $\tilde{n}_{\rm min}({\bf r})$; in an experiment, Σ is the set of all vertex positions for which the fluorescence signal is well above the noise level. In figure 3, Σ is shown as a simple connected region but one can consider more complex geometries. If $\Sigma = \Omega$, the fluorescence signal can be measured in the whole domain of interest. We say that in this case we have access to complete data. This situation occurs when a significant concentration of the fluorescent molecules are present everywhere in Ω.

In this section, we discuss inversion of (16) with complete data. Then in section 6 we sketch some approaches to reconstruction when complete data are not available. We begin by noting that (16*a*) is equivalent to the star transform [23] of a medium with the attenuation coefficient $\bar{\mu}({\bf r})$. The dimensionless concentration of fluorophores $\tilde{n}({\bf r})$ plays the role of the contrast of the scattering coefficient. We can therefore use the methods described in [23] to reconstruct simultaneously $\bar{\mu}({\bf r})$ and $\xi({\bf r})$; the dimensionless concentration is then trivially computed as $ \newcommand{\e}{{\rm e}} \tilde{n}({\bf r}) = \exp(-\xi({\bf r}))$.

Consider the three-ray geometry shown in figure 2. We then define the vector coefficients ${\bf a}_{kl}$ ($k, l=1, 2, 3$) as shown in the matrix

18 \begin{align*} \newcommand{\e}{{\rm e}} \displaystyle \label{a_N=3} \left[\begin{array}{@{}ccc@{}} 0 &amp; \sigma_1 \hat{\bf u}_1 + \sigma_2 \hat{\bf u}_2 &amp; \sigma_1 \hat{\bf u}_1 + \sigma_3 \hat{\bf u}_3 \nonumber \\ \sigma_1 \hat{\bf u}_1 + \sigma_2 \hat{\bf u}_2 &amp; 0 &amp; \sigma_2 \hat{\bf u}_2 + \sigma_3 \hat{\bf u}_2 \nonumber \\ \sigma_1 \hat{\bf u}_1 + \sigma_3 \hat{\bf u}_3 &amp; \sigma_2 \hat{\bf u}_2 + \sigma_3 \hat{\bf u}_2 &amp; 0 \end{array}\right]. \nonumber \end{align*}

Here the scalar coefficients $\sigma_k$ satisfy the condition

19 \begin{align*} \newcommand{\e}{{\rm e}} \displaystyle \label{sig_cond} \sigma_1 \hat{\bf u}_1 + \sigma_2 \hat{\bf u}_2 + \sigma_3 \hat{\bf u}_3 = 0 \ . \nonumber \end{align*}

Such coefficients can always be found since three vectors on a plane can not be linearly-independent. We then take the following linear combination of the symmetric functions $\phi_{kl}^{(+)}({\bf r})$ (by symmetry, we mean here the property $\phi_{kl}^{(+)} = \phi_{lk}^{(+)}$):

20 \begin{align*} \newcommand{\e}{{\rm e}} \displaystyle \label{Phi_phi_p} {\boldsymbol \Phi}^{(+)}({\bf r}) = \frac{1}{2} \sum_{k,l=1}^3 {\bf a}_{kl} \phi_{kl}^{(+)}({\bf r}) \ . \nonumber \end{align*}

It is easy to see that the resulting function ${\boldsymbol \Phi}^{(+)}({\bf r})$ satisfies

21 \begin{align*} \newcommand{\e}{{\rm e}} \displaystyle \sum_{k=1}^3 \sigma_k \hat{\bf u}_k I^{(+)}_k({\bf r}) = {\boldsymbol \Phi}^{(+)}({\bf r}) \ . \nonumber \end{align*}

We now use the property $-\nabla \cdot {\bf u}_k I_k^{(+)}({\bf r}) = \bar{\mu}({\bf r})$ to find that

22 \begin{align*} \newcommand{\e}{{\rm e}} \displaystyle \label{rec_mubar} \bar{\mu}({\bf r}) = -\frac{1}{\sigma_1 + \sigma_2 + \sigma_3}\nabla \cdot {\boldsymbol \Phi}^{(+)}({\bf r}) \ . \nonumber \end{align*}

Thus, we have a local reconstruction formula for the spectrally-averaged attenuation coefficient $\bar{\mu} = (\mu_e + \mu_f)/2$. It is not surprising that this formula utilizes the symmetric data functions $\phi_{kl}^{(+)}({\bf r})$ or a linear combination thereof ${\boldsymbol \Phi}^{(+)}({\bf r})$, as defined in (20). Indeed, the symmetric data points are obtained by combining the measurements in which the photon travels through the medium in both possible directions (for a given broken ray). When the photon travels in one of these directions, it is attenuated at the rate $\mu_e$ in the first segment of the broken ray and $\mu_f$ in the second segment. When the direction of propagation is reversed, the rate of attenuation is $\mu_f$ in the first segment and $\mu_e$ in the second segment. By adding the two measurements together, we are effectively performing an average of the attenuation coefficient.

Since we have assumed here that complete data are available, we can reconstruct $\bar{\mu}({\bf r})$ everywhere in Ω. We can then use this result to compute the ray integrals $I_k^{(+)}({\bf r})$ according to (16*a*). Once this is done, we can obtain $\xi({\bf r})$ from any of the equations in (15*a*). This procedure yields a complete reconstruction of $\tilde{n}({\bf r})$. However, to obtain the spectrally-resolved intrinsic attenuation coefficients $\mu^{(0)}_e({\bf r})$ and $\mu^{(0)}_f({\bf r})$, we also need to know the spectral difference $\Delta({\bf r})$ of the attenuation coefficients. We can use the anti-symmetric data functions $\phi_{kl}^{(-)}({\bf r})$ to reconstruct $\Delta({\bf r})$ by inverting (15*b*). The latter equation is similar to the star transform (15*a*) but has a different sign and also does not contain the term $\xi({\bf r})$. Therefore, inverting (15*b*) is slightly different from inverting (15*a*).

Recall that in order to derive a local inversion formula for (15*a*), we need to find a set of vector coefficients ${\bf a}_{kl}$ that satisfy the following four conditions [23]: (i) ${\bf a}_{kl} = {\bf a}_{lk}$; (ii) ${\bf a}_{kk}=0$; (iii) $\sum_l {\bf a}_{kl} = \sigma_k \hat{\bf u}_k$; and (iv) $\sum_{kl} {\bf a}_{kl} = 0$. For the conditions (i) and (iv) to be consistent, the scalar coefficients $\sigma_k$ must satisfy $\sum_k \sigma_k \hat{\bf u}_k = 0$. If the above four conditions hold, we have $\frac{1}{2}\sum_{kl}{\bf a}_{kl} (x_k + x_l + y) = \sum_k \sigma_k \hat{\bf u}_k x_k$, where *x*_*k*_ and *y* are arbitrary numbers. This property allows us to invert the star transform (15*a*) locally.

In order to invert (15*b*), we need to find a set of coefficients ${\bf b}_{kl}$ such that $\frac{1}{2}\sum_{kl}{\bf b}_{kl} (x_k - x_l) = \sum_k \sigma_k \hat{\bf u}_k x_k$. It is easy to see that ${\bf b}_{kl}$ must satisfy only three conditions, namely, (i) ${\bf b}_{kl} = -{\bf b}_{lk}$; (ii) ${\bf b}_{kk}=0$; and (iii) $\sum_l {\bf b}_{kl} = \sigma_k \hat{\bf u}_k$. We note that the condition (iv) $\sum_{kl}{\bf b}_{kl} = 0$ also holds but is a consequence of (i) rather than an independent condition. It also follows from (i) and (iii) that $\sigma_k$ must still satisfy $\sum_k \sigma_k \hat{\bf u}_k = 0$. For the three-ray geometry considered here, the following matrix of coefficients ${\bf b}_{kl}$ satisfy all stated conditions:

23 \begin{align*} \newcommand{\e}{{\rm e}} \displaystyle \label{b_N=3} \left[\begin{array}{@{}ccc@{}} 0 &amp; -\sigma_1 \hat{\bf u}_1 &amp; 0 \nonumber \\ \sigma_1 \hat{\bf u}_1 &amp; 0 &amp; \sigma_3 \hat{\bf u}_3 \nonumber \\ 0 &amp; -\sigma_3 \hat{\bf u}_3 &amp; 0 \end{array}\right]. \nonumber \end{align*}

The coefficients $\sigma_k$ in this matrix are the same as in matrix (23) and determined from (19). We note that (23) does not contain $\hat{\bf u}_2$ explicitly but $\hat{\bf u}_2$ is not linearly independent of $\hat{\bf u}_1$ and $\hat{\bf u}_3$. In fact, (23) is not a unique choice of coefficients; the conditions stated above for the elements ${\bf b}_{kl}$ can be written equivalently as ${\bf b}_{12} = {\bf b}_{21} + \sigma_2 \hat{\bf u}_2$ and ${\bf b}_{13} + {\bf b}_{23} = \sigma_3\hat{\bf u}_3$. In deriving (23), we have made the choice ${\bf b}_{13}=0$. However, we can also take ${\bf b}_{23} = 0$, which results in the matrix

24 \begin{align*} \newcommand{\e}{{\rm e}} \displaystyle \label{b_N=3_prime} \left[\begin{array}{@{}ccc@{}} 0 &amp; \sigma_2 \hat{\bf u}_2 &amp; \sigma_3 \hat{\bf u}_3 \nonumber \\ -\sigma_2 \hat{\bf u}_2 &amp; 0 &amp; 0 \nonumber \\ -\sigma_3 \hat{\bf u}_3 &amp; 0 &amp; 0 \end{array}\right]. \nonumber \end{align*}

There are other possible choices in which all six off-diagonal elements of the matrix are non-zero and all three vectors $\hat{\bf u}_1$, $\hat{\bf u}_2$ and $\hat{\bf u}_3$ are explicitly present. Noting this will restore the symmetry, which is seemingly lost in (23). Indeed, there is nothing special about the vector $\hat{\bf u}_2$, which is not present explicitly in (23) or $\hat{\bf u}_1$, which is not present in (24).

We now define a combination of the anti-symmetric data functions according to

25 \begin{align*} \newcommand{\e}{{\rm e}} \displaystyle \label{Phi_phi_m} {\boldsymbol \Phi}^{(-)}({\bf r}) = \frac{1}{2} \sum_{k,l=1}^3 {\bf b}_{kl} \phi_{kl}^{(-)}({\bf r}) \ . \nonumber \end{align*}

For the matrix of coefficients defined in (23), or for any other such matrix with any choice for the two indices, the right-hand side of (25) is of the form $-\sum_{k=1}^3 \sigma_k \hat{\bf u}_k I_k$. Therefore, (25) is inverted by taking the divergence. Based on this observation, we find that the reconstruction formula for the spectral difference of the attenuation coefficients, which is of the form

26 \begin{align*} \newcommand{\e}{{\rm e}} \displaystyle \label{rec_Delta} \Delta({\bf r}) = -\frac{1}{\sigma_1 + \sigma_2 + \sigma_3}\nabla \cdot {\boldsymbol \Phi}^{(-)}({\bf r}) \nonumber \end{align*}

in complete analogy with (22). We thus have obtained direct reconstruction formulas for $\bar{\mu}({\bf r})$, $\Delta({\bf r})$ and $\xi({\bf r})$. From these results, we can easily obtain $\mu_e({\bf r})$, $\mu_f({\bf r})$ and $\tilde{n}({\bf r})$ by using (17) and (11*a*). If we know the absorption cross section and quantum efficiency of a singe fluorophore, we can compute the physical number density of the fluorescent molecules $n({\bf r})$ and, once the latter is known, we can find the intrinsic attenuation coefficients of the medium $\mu^{(0)}_e({\bf r})$ and $\mu^{(0)}_f({\bf r})$ from (19). We thus have obtained direct reconstruction formulas for all optical properties of the medium.

## 6. Inversion with incomplete data

If complete data are not available, we can still reconstruct the total attenuation coefficients $\mu_e({\bf r})$ and $\mu_f({\bf r})$ for ${\bf r} \in \Sigma$. To this end, we simply use equations (22) and (26), which are local and do not require complete data, to find $\bar{\mu}({\bf r})$ and $\Delta({\bf r})$, and then use $\mu_e = \bar{\mu} + \frac{1}{2}\Delta$, $\mu_f = \bar{\mu} - \frac{1}{2}\Delta$. In some cases this provides sufficient information and no further image reconstruction is necessary. However, it is not possible to find $\xi({\bf r})$ from the above result in the absence of complete data. The reason is that we can not compute the ray integrals $I_k^{(+)}({\bf r})$ according to (16*a*). Consequently, we can not obtain $\xi({\bf r})$ from (15*a*). One possible solution to this problem is to introduce some assumptions about the spectral dependence of the intrinsic attenuation. For example, we may know that $\mu^{(0)}_e({\bf r}) / \mu^{(0)}_f({\bf r}) = \kappa$, where *κ* is a position-independent constant (which depends on the frequencies $\omega_e$ and $\omega_f$). Indeed, we have from (3) and the above assumption

27 \begin{align*} \newcommand{\e}{{\rm e}} \displaystyle \kappa \mu^{(0)}_f({\bf r}) + \sigma_e n({\bf r}) = \mu_e({\bf r}) , \ \ \mu^{(0)}_f({\bf r}) + \sigma_f n({\bf r}) = \mu_f({\bf r}) , \nonumber \end{align*}

where the functions $\mu_e({\bf r})$ and $\mu_f({\bf r})$ are known in Σ. From this we find

28 \begin{align*} \newcommand{\e}{{\rm e}} \displaystyle n({\bf r}) = \frac{\mu_e({\bf r}) - \kappa \mu_f({\bf r})}{\sigma_e - \kappa \sigma_f} , \ \ \mu^{(0)}_f({\bf r}) = \frac{\sigma_e\mu_f({\bf r}) - \sigma_f\mu_e({\bf r})}{\sigma_e - \kappa \sigma_f} \ . \nonumber \end{align*}

The above condition on the spectral dependence is equivalent to the assumption that the medium contains two chemical species of molecules or macroscopic particles that are responsible for the attenuation (one fluorescent and one not), and that the optical properties of the medium are fully described by two mathematically-independent density functions. In samples with complex chemical and structural composition, this might not hold. Since different species of absorbers can have different optical spectra, the assumption $\mu^{(0)}_e({\bf r}) / \mu^{(0)}_f({\bf r}) = \kappa$ may not be justified. In this case, additional spectral measurements could be employed to access the missing information. For instance, we have assumed that the light recorded by the detectors has passed through a spectral filter that eliminates light at the excitation frequency $\omega_e$. However, we can also employ spectrally-resolved detectors that can measure the light intensities at the excitation and fluorescence frequencies separately. Then, if the medium is weakly scattering, we can use intensity measurements at the excitation frequency to reconstruct $\mu_e({\bf r})$ everywhere in Ω. This does not require an additional scan, only a spectrally-resolved measurement of intensity.

Finally, we note that knowledge of $\mu_e({\bf r})$ everywhere in Ω is insufficient to reconstruct $n({\bf r})$. We also need to know $\mu_f({\bf r})$ in Ω. It appears that the only feasible approach to recover this function is to perform an additional scan using $\omega_f$ as the excitation frequency. Fluorescence in this case is not excited and the signal at $\omega_f$ is due to single scattering. If this additional scan is possible, then the ray integrals $I_k^{(+)}({\bf r})$ can be computed according to (16*a*) and we can further solve for $\xi({\bf r})$ just as in the case of complete data.

## 7. Summary

We have shown that the BRT is non-reciprocal if the photon can change its frequency at the vertex of the broken ray. The non-reciprocity provides an opportunity to gain additional information about the medium by interchanging the source and detector in each measurement. We note that the image reconstruction methods described in this paper are based on the original idea of Katsevich and Krylov [22], and are direct and spatially-local.

We have discussed applications of non-reciprocity to tomographic optical imaging of a medium containing a fluorescent contrast agent. In this case, the quantities of interest are the number density of the fluorophores and the intrinsic attenuation coefficients of the medium at the excitation and fluorescence frequencies. All three functions can be reconstructed simultaneously and independently by inverting the three-ray star transform of the medium (consisting of three broken rays with a common vertex and some common edges, as shown in figure 2), assuming that a complete data set is available. Physically, this condition means that there is a significant concentration of the fluorescent agent in the region of interest. If this condition is not satisfied, reconstructions can still be performed, as can a complete reconstruction with the use of additional spectrally-resolved measurements, as is discussed in section 6.

Note that the formalism presented above does not make any assumption about the angles of the broken rays (except if applied to Compton scattering of x-rays). As a result, both back-scattering and transmission measurement geometries can be implemented. The back-scattering measurement geometry is important for *in vivo* imaging of mesocopic systems.

The method proposed in this paper is applicable to single scattering x-ray tomography in which the photon energy changes due to Compton scattering. In this case, complete data will almost always be available. In order to apply the reconstruction methods described in this paper to Compton scattering, it is important to use only broken rays with the same angle to guarantee that the Compton energy shifts are always the same. The symmetric three-ray star geometry satisfies this condition: all scattering angles in this case are equal to $2\pi/3$. We note that the formalism presented here enables a new approach to dual-energy x-ray CT that could potentially overcome some of the limitations of this technique due to hardware, software, or dose constraints [25]. Indeed, for a Compton scattering angle of $2\pi/3$, the energy separation between the primary (excitation) and scattered radiation is helpful for improved tissue differentiation based on simultaneous reconstruction of tissue properties at two energies. At the same time, this method can be realized with single-energy exposure and single-energy detection. Moreover, the spectral distribution of the detected scattered radiation can be much narrower than that of the detected lower-energy radiation conventionally used in dual-energy CT. This could be useful for ameliorating the artifacts associated with beam hardening.
